# Informal and Professional Awareness of Nonsuicidal Self-Injury: Clinical and Functional Correlates Among Arab Female University Students

**DOI:** 10.3390/jcm15208166

**Published:** 2026-10-20

**Authors:** Sami Hamdan, Eyad Hallaq

**Affiliations:** 1 School of Behavioral Sciences, The Academic College of Tel Aviv-Yaffo, Tel Aviv-Yaffo 6818211, Israel; 2 Department of Psychology, Al-Quds University, East Jerusalem, Palestine; drhallaq@yahoo.com

**Keywords:** nonsuicidal self-injury, NSSI, clinical assessment, recipient awareness, professional awareness, depressive symptoms, NSSI functions, university students

## Abstract

**Background/Objectives:** Clinical assessment of nonsuicidal self-injury (NSSI) typically documents the behavior, its functions, and current safety, but less attention has been given to who is aware of the NSSI. Informal and professional recipients occupy different roles in support, assessment, and referral, yet disclosure research often combines them. We examined whether clinical and functional correlates differed by recipient type among Arab female university students. **Methods:** A cross-sectional online survey included 8320 participants, of whom 2396 reported lifetime NSSI. Recipient awareness was classified as no awareness among five assessed categories (*n* = 812), informal-only awareness by a friend, family member, or partner (*n* = 824), or professional awareness by a mental health professional or physician, with or without informal awareness (*n* = 760). Analyses included multinomial logistic regression, adjusted probabilities, generalized estimating equations, and sensitivity analyses. **Results:** Depressive symptoms and NSSI method versatility were more strongly associated with professional than informal awareness (ORs for the recipient-class slope differences = 1.54 and 1.32, respectively). Interpersonal-function endorsement was associated with both informal and professional awareness and distinguished professional from informal-only awareness (RRR = 1.25). Intrapersonal-function endorsement did not retain an independent association after adjustment. **Conclusions:** Informal-only and professional awareness were associated with different concurrent NSSI profiles. In clinical assessment, asking who knows about the NSSI, how awareness arose, and what happened afterward may add useful context to formulation. Professional awareness should not be taken as evidence that adequate assessment or care occurred. The cross-sectional data do not establish disclosure, help-seeking, treatment engagement, or temporal sequence.

## 1. Introduction

Nonsuicidal self-injury (NSSI) is the deliberate destruction of body tissue without suicidal intent and for purposes that are not socially sanctioned. Clinical assessment requires more than establishing that NSSI has occurred. Contemporary guidance for self-harm recommends psychosocial assessment of the reasons and functions of self-harm, current needs and safety, available support, and the appropriate involvement of family or carers [1]. Although this guidance addresses self-harm more broadly, these assessment principles are also relevant to NSSI.

Functional models distinguish automatic and social reinforcement processes [2], while the Inventory of Statements About Self-Injury organizes a broader range of functions into intrapersonal and interpersonal domains [3]. Intrapersonal functions, particularly regulation of intense affect, are most commonly endorsed. Interpersonal functions are less prevalent but place NSSI within a relational context [4].

How NSSI functions and how it becomes known to another person are related but distinct questions. Behavior used primarily to regulate internal states may later be disclosed, observed, or identified during assessment, whereas NSSI with interpersonal functions may remain unknown. Awareness can arise through direct disclosure, visible injury, medical care, clinical questioning, or information from a third party. Disclosure is narrower because it entails intentional communication of previously concealed information [5,6,7,8]. Here, professional awareness means only that a mental health professional or physician was reported to know about the NSSI. It does not establish how awareness arose, whether an NSSI- or suicide-focused assessment occurred, or whether treatment was initiated or sustained.

Informal and professional recipients occupy different roles. Friends, partners, and family members are common recipients of NSSI disclosure, and decisions to tell may be shaped by stigma, anticipated rejection, concern about burdening others, and loss of control over the information [6,9]. Supportive reactions may facilitate further communication, whereas alarmed, dismissive, punitive, or controlling responses may inhibit it [10,11].

Longitudinal and qualitative evidence indicates that disclosure to peers, parents, and professionals should not be treated as interchangeable events [12,13,14]. Professional awareness creates an opportunity to assess NSSI, co-occurring symptoms, suicidal thoughts and behaviors, and treatment needs, but it does not demonstrate that such assessment occurred.

Empirical findings on NSSI functions and disclosure have been inconsistent. Among Canadian university students, interpersonal influence differentiated those who disclosed from those who did not [15]. An Australian study identified marking distress and anti-dissociation as relevant functions [16], whereas research with U.S. undergraduates linked disclosure to broader intrapersonal motives, suicide risk, perceived support, and lower depressive symptoms [17]. Variation in measures and covariate structure may partly account for these differences. Recipient composition is another possibility: a binary disclosed-versus-undisclosed outcome can combine people with markedly different relational and clinical roles. Distinguishing informal from professional awareness permits a direct test of recipient-specific patterns.

Two empirical questions follow from this distinction. First, does NSSI known to an assessed recipient differ from NSSI unknown to all five assessed recipient categories? Interpersonal functions are relevant to this comparison because several items concern communication, support, influence, and attachment, although their conceptual proximity to social awareness requires caution. Second, what distinguishes NSSI known only to informal recipients from NSSI known to a mental health professional or physician? We examined depressive symptoms and NSSI method versatility as potential differentiating correlates. These analyses concern concurrent profiles, not a sequence from concealment to disclosure to professional care. Method count is therefore interpreted as versatility rather than medical severity.

Arab female university students remain underrepresented in research on NSSI awareness and disclosure. Studies of mental health help-seeking in Arab populations describe reliance on informal support alongside concerns about privacy, confidentiality, stigma, trust, and service acceptability [18,19]. NSSI and substantial psychological distress have also been documented among Arab women in higher education [20]. These observations establish the population context for the present analysis, but they do not support a cultural explanation of the associations because cultural mechanisms were not measured and no gender, ethnic, or cross-national comparison group was included.

We examined three mutually exclusive recipient configurations: no awareness among the five assessed categories, informal-only awareness involving a friend, family member, or partner, and professional awareness involving a mental health professional or physician, with or without informal awareness. The primary objective was to compare depressive symptoms, NSSI frequency and method versatility, and intrapersonal and interpersonal functions across these configurations. We expected interpersonal functions to distinguish both awareness groups from no assessed awareness, and depressive symptoms and method versatility to show stronger associations with professional than informal-only awareness. These hypotheses concerned cross-sectional associations, not prediction, mediation, or temporal progression.

## 2. Materials and Methods

### 2.1. Study Design and Participants

This cross-sectional online study used nonprobability sampling. Recruitment took place from June 2024 through February 2026 through WhatsApp, Facebook, Instagram, student groups, email lists, peer-to-peer circulation, and snowball referral. Eligible participants were Arab women born in Israel, aged at least 18 years, and enrolled in a university, college, or other higher-education institution in Israel or abroad. Because recruitment did not draw from a defined institutional sampling frame, the number of people exposed to the invitation was unknown, and a conventional response rate could not be calculated.

Approximately 9369 consenting and eligible individuals entered the survey. Of these, 1049 did not complete the questionnaire and were excluded before analysis; most stopped during the initial demographic section. The information provided by these respondents was generally insufficient for a meaningful comparison with completers. All 8320 eligible completed questionnaires were retained. Lifetime NSSI was endorsed by 2396 participants (28.8%), who formed the focal analytic subgroup. This proportion describes the recruited sample and should not be interpreted as a population prevalence estimate.

Participants in the lifetime-NSSI subgroup were 18 to 45 years old (M = 22.12, SD = 3.67). Among the 2396 participants, 812 (33.9%) reported no awareness among the five assessed recipient categories, 824 (34.4%) reported informal-only awareness, and 760 (31.7%) reported professional awareness by a mental health professional or physician. Within the professional-awareness group, 197 reported professional-only awareness and 563 reported both informal and professional awareness.

### 2.2. Procedure, Ethics, and Participant Safety

The study was approved by the Institutional Review Board of the Academic College of Tel Aviv-Yaffo (IRB No. 2024-228). The information sheet stated that the questionnaire contained sensitive questions about self-injury and suicidal thoughts, and participants were free to skip any items they preferred not to answer. Participants were informed that the survey did not constitute clinical monitoring or treatment. Because responses were anonymous and no contact information was collected, individualized outreach or follow-up was not possible. At the end of the survey, participants could access information about available emotional and professional support.

### 2.3. Measures

#### 2.3.1. Demographic and Treatment History

Participants reported age, academic stage, relationship status, living arrangement, region, locality type, religious affiliation, religiosity, and previous mental health treatment. Relationship status was coded as partnered or married versus all other categories. A binary treatment history item asked whether the participant had ever received psychological or psychiatric treatment or counseling.

#### 2.3.2. Nonsuicidal Self-Injury Characteristics

Lifetime NSSI and approximate lifetime frequency were assessed for 10 methods using selected items derived from the Nonsuicidal Self-Injury Assessment Tool (NSSI-AT) [21]: cutting, burning, self-hitting, self-biting, severe scratching, interference with wound healing, rubbing the skin against a rough surface, hair pulling intended to cause injury, severe pinching, and another intentional form of self-injury. Before the method items, participants were instructed to count a behavior only when it was performed intentionally to injure themselves and without an intention to die; accidental acts were not counted. The same intentional self-injury criterion applied to all listed methods. Thus, hair pulling was counted only when intended to cause injury, and interference with wound healing was counted only when performed as intentional self-injury rather than as an incidental act. Suicidal thoughts and suicidal behavior were assessed separately and were not included in NSSI frequency or method versatility. Participants provided an estimated lifetime count for each method, recording zero for methods they had never used. Method versatility was operationalized as the number of methods with a count greater than zero. Total lifetime frequency was the sum of method counts and was transformed as ln(1 + frequency) for regression because of marked right skew. A separate item assessed whether an NSSI episode had required professional medical attention; because such care can itself create physician awareness, this item was not treated as a severity covariate in the primary models.

#### 2.3.3. NSSI Functions

Ten function items derived from the ISAS framework were translated from English into Arabic and then back-translated into English following established cross-cultural translation procedures [3,22]. Discrepancies between the source and back-translated versions were reviewed and resolved before administration. Five intrapersonal items assessed relief from overwhelming distress, self-punishment, reduction in numbness or detachment, making psychological pain concrete, and reduction in suicidal thoughts or prevention of suicidal action. Five interpersonal items assessed communication of distress, obtaining care or support, conveying the seriousness of distress, influencing an interpersonal situation, and preventing rejection or abandonment. Responses ranged from 1 (not at all) to 5 (extremely). Mean scores were calculated separately for the intrapersonal and interpersonal item sets. These abbreviated item sets do not constitute the complete ISAS subscales. The complete English rendering of the 10 item contents used in this study is provided in Supplementary Table S4. Internal consistency was adequate for the intrapersonal (α = 0.838) and interpersonal (α = 0.827) scores.

#### 2.3.4. Depressive Symptoms

Depressive symptoms during the preceding two weeks were assessed with the eight-item Patient Health Questionnaire (PHQ-8) [23], using validated Arabic wording [24]. Items were rated from 0 (not at all) to 3 (nearly every day). Total scores ranged from 0 to 24; higher scores indicated greater symptom severity. Internal consistency in the present sample was high (α = 0.897).

#### 2.3.5. Recipient-Specific NSSI Awareness

Participants with lifetime NSSI indicated if the behavior had become known to a friend, family member, spouse or partner, therapist or mental health professional, and physician. The instructions indicated that ‘yes’ should be chosen if the participant directly told the recipient or if the recipient learned through other means. Informal awareness included awareness from friends, family, or partners. Professional awareness comprised awareness among mental health professionals or physicians. The primary outcome distinguished no assessed awareness, informal-only awareness, and professional awareness with or without informal awareness. These are nominal recipient configurations, not ordered stages. No assessed awareness is not equivalent to complete concealment, and professional awareness does not establish disclosure, help-seeking, or treatment engagement.

### 2.4. Statistical Analysis

The analyses were restricted to participants who reported a lifetime history of NSSI. Descriptive statistics were generated for demographic details, NSSI features, depressive symptoms, NSSI functions, and recipient-awareness status. Continuous variables were compared across awareness groups using Welch’s ANOVA or the Kruskal–Wallis test as appropriate, while categorical variables were analyzed with Pearson’s chi-square tests. Effect sizes were presented as η^2^, ε^2^, or Cramér’s V, depending on the test. Holm-adjusted *p* values were used for pairwise comparisons.

The primary outcome consisted of three nominal categories: no assessed awareness, informal-only awareness, and professional awareness (with or without informal awareness). Cross-sectional associations with awareness status were estimated using multinomial logistic regression. No assessed awareness was the reference category, and the model was re-parameterized to obtain the direct professional-versus-informal-only contrast. The prespecified covariates were age, partnered or married status, depressive symptoms, lifetime NSSI frequency, method versatility, and intrapersonal and interpersonal NSSI functions. Lifetime NSSI frequency was log-transformed because of marked positive skew. Continuous covariates were standardized within the lifetime-NSSI sample, so each relative risk ratio represents the association corresponding to a one-standard-deviation higher value. HC3 heteroskedasticity-robust standard errors were used, and results are reported as relative risk ratios with 95% confidence intervals. Average adjusted probabilities were calculated to aid interpretation. The model was used to characterize associations among recipient configurations, not for individual-level clinical prediction.

To compare the same covariate across informal and professional awareness directly, we also fitted generalized estimating equation logistic models. Each participant contributed two correlated binary outcomes: awareness by any informal recipient and awareness by any professional recipient. Models included recipient class, the study covariates, and recipient-class-by-covariate interactions. An exchangeable working correlation structure and robust standard errors accounted for within-participant dependence. Holm adjustment was applied to the focal interaction tests for depressive symptoms, method versatility, and interpersonal functions.

Sensitivity analyses examined whether the focal associations were robust to alternative specifications. Previous mental health treatment was included because it partly indexes opportunity for professional contact and may lie on the same contact process as the professional-awareness outcome; it was, therefore, not treated as a conventional confounder in the primary model. Informal awareness was also redefined to include only friend and family awareness, excluding spouse or partner awareness. A four-category multinomial model distinguished no assessed awareness, informal-only awareness, professional-only awareness, and combined informal and professional awareness. Because lifetime NSSI frequency and method versatility were correlated, additional models entered each separately and concurrently. Following peer review, three additional post hoc sensitivity analyses were conducted: (1) a paired GEE model directly compared associations with mental-health-professional awareness and physician awareness; (2) the focal multinomial and paired GEE analyses were repeated after excluding participants who reported that NSSI had required professional medical attention; and (3) the interpersonal-function score was decomposed into a communication/signaling pair (communication of distress and conveying the seriousness of distress) and the remaining three interpersonal items, which were entered simultaneously. These post hoc analyses served as exploratory robustness checks and did not change the primary estimands.

Missing data were handled using analysis-specific complete-case analysis. Participants were excluded only from analyses for which one or more required variables were missing; no values were imputed. The analytic sample size is reported for each model. All tests were two-sided with α = 0.05.

### 2.5. Use of Generative Artificial Intelligence in Manuscript Preparation

Google Gemini (Google LLC, Mountain View, CA, USA) was used during manuscript preparation and revision to assist with language editing, organization, clarity, and refinement of the manuscript’s presentation. Its use was limited to editorial assistance. All AI-assisted content was critically reviewed and edited by the authors, who take full responsibility for the accuracy, integrity, and final content of the manuscript.

## 3. Results

### 3.1. Recipient Configurations and Unadjusted Group Differences

Among the 2396 participants with lifetime NSSI, 1584 (66.1%) reported awareness by at least one of the five assessed recipient categories. Informal awareness was reported by 1387 participants, professional awareness by 760, and both by 563. Age, relationship status, and previous treatment differed little across the three mutually exclusive configurations (Table 1). Mean depressive symptoms, method versatility, and intrapersonal and interpersonal function scores were highest in the professional-awareness group.

Intrapersonal-function scores differed between groups, Welch’s F(2, 1590.58) = 15.06, *p* < 0.001, η^2^ = 0.012, with higher scores in the professional-awareness group compared to the no-awareness group (d = 0.22, Holm-adjusted *p* < 0.001) and the informal-only group (d = 0.26, Holm-adjusted *p* < 0.001). The no-awareness and informal-only groups did not differ significantly (d = 0.04, Holm-adjusted *p* = 0.426). Interpersonal function scores exhibited an even larger difference, Welch’s F(2, 1592.36) = 63.84, *p* < 0.001, η^2^ = 0.051; all pairwise comparisons remained significant after Holm correction.

### 3.2. Primary Multinomial Model

The multinomial model differed from the intercept-only model, likelihood-ratio χ^2^(14) = 205.51, *p* < 0.001. Overall separation among recipient configurations was limited (McFadden R^2^ = 0.039; AIC = 5088.12; BIC = 5180.63). Multicollinearity was not marked; the maximum variance inflation factor was 2.89. Coefficients are interpreted as adjusted cross-sectional associations rather than as measures of predictive performance.

The adjusted relative risk ratios for all planned contrasts are presented in Table 2.

A one-standard-deviation higher interpersonal-function score was associated with a higher relative likelihood of informal-only rather than no assessed awareness (RRR = 1.39, 95% CI [1.25, 1.54], *p* < 0.001) and of professional rather than no assessed awareness (RRR = 1.73, 95% CI [1.55, 1.93], *p* < 0.001). It also distinguished professional from informal-only awareness (RRR = 1.25, 95% CI [1.12, 1.38], *p* < 0.001). Intrapersonal functions were not independently associated with any planned contrast after adjustment (all *p* ≥ 0.259).

Depressive symptoms showed a different pattern across recipient configurations. A one-standard-deviation higher PHQ-8 score was associated with a lower relative likelihood of informal-only rather than no assessed awareness (RRR = 0.81, 95% CI [0.72, 0.92], *p* < 0.001), but a higher relative likelihood of professional rather than no assessed awareness (RRR = 1.14, 95% CI [1.01, 1.30], *p* = 0.036). The direct professional-versus-informal-only contrast was stronger (RRR = 1.41, 95% CI [1.25, 1.60], *p* < 0.001).

Method versatility did not distinguish informal-only from no assessed awareness (RRR = 0.94, 95% CI [0.80, 1.11], *p* = 0.494). By contrast, a one-standard-deviation higher method-versatility score was associated with a greater relative likelihood of professional versus no assessed awareness (RRR = 1.34, 95% CI [1.13, 1.60], *p* < 0.001) and professional versus informal-only awareness (RRR = 1.42, 95% CI [1.19, 1.70], *p* < 0.001).

When lifetime frequency and method versatility were entered together, log lifetime frequency had inverse coefficients in the contrasts involving professional awareness. Frequency was not associated with professional awareness in models that omitted method versatility. We therefore interpret the sign reversal as a conditional suppression pattern, not as evidence that greater lifetime frequency is protective.

### 3.3. Adjusted Probabilities and Formal Recipient-Class Comparisons

**Figure 1:**
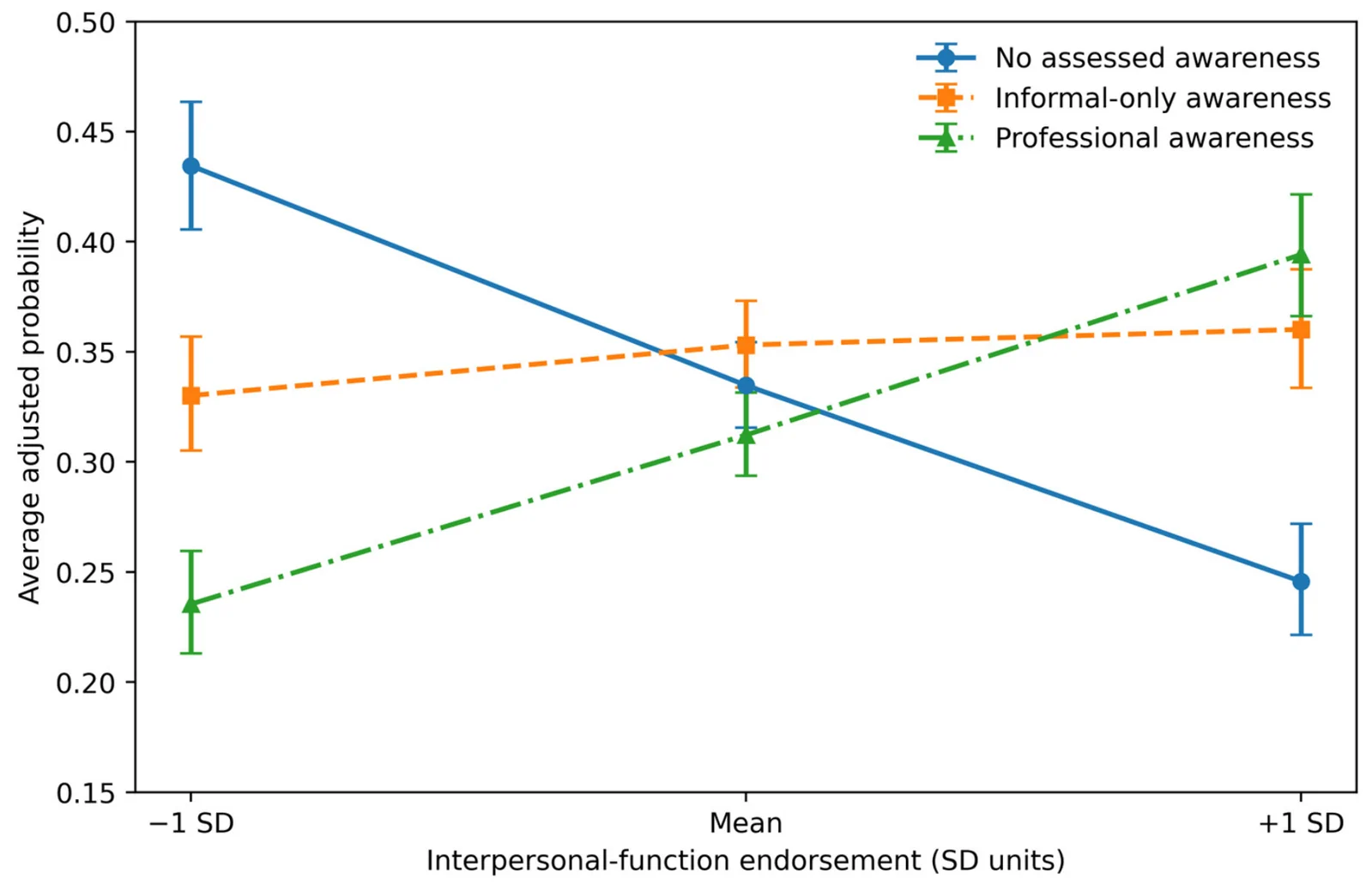
Adjusted probabilities of recipient-awareness configurations across interpersonal-function endorsement. Note. Average adjusted probabilities are shown at 1 SD below the mean, the mean, and 1 SD above the mean of interpersonal-function endorsement. Estimates are adjusted for age, relationship status, PHQ-8 symptoms, log lifetime NSSI frequency, method versatility, and intrapersonal functions. Error bars represent 95% confidence intervals.

Adjusted probabilities illustrated these contrasts. Across interpersonal-function scores from 1 SD below to 1 SD above the mean, the probability of no assessed awareness decreased from 43.5% to 24.6%, while the probability of professional awareness increased from 23.5% to 39.4%; informal-only awareness changed from 33.0% to 36.0% (Figure 1). Across the corresponding PHQ-8 range, informal-only awareness decreased from 40.6% to 28.4% and professional awareness increased from 26.4% to 36.4%. Across the method-versatility range, professional awareness increased from 25.2% to 38.5%. These are model-based adjusted probabilities, not estimates of temporal transition between recipient categories.

The paired GEE models confirmed that the associations of depressive symptoms and method versatility differed by recipient class (Table 3). For depressive symptoms, the OR was 0.83 for informal awareness and 1.28 for professional awareness; the professional-versus-informal slope ratio was OR = 1.54, 95% CI [1.34, 1.75], Holm-adjusted *p* < 0.001. Method versatility was not associated with informal awareness (OR = 1.05) but was associated with professional awareness (OR = 1.38); the slope ratio was OR = 1.32, 95% CI [1.09, 1.58], Holm-adjusted *p* = 0.007. Interpersonal functions were associated with both informal awareness (OR = 1.34) and professional awareness (OR = 1.47), but the slopes did not differ significantly (OR = 1.09, 95% CI [0.98, 1.22], Holm-adjusted *p* = 0.128).

### 3.4. Sensitivity Analyses

The focal multinomial estimates changed little after adjustment for previous mental health treatment and after redefining informal awareness to include friends and family only. In the partner-excluded GEE, depressive symptoms and method versatility were more strongly associated with professional than with informal awareness, whereas interpersonal functions were positively associated with both recipient classes (Tables S1 and S3).

The four-category model clarified heterogeneity within the pooled professional-awareness configuration. Compared with no assessed awareness, depressive symptoms were most strongly associated with professional-only awareness (RRR = 1.47, 95% CI [1.21, 1.80]), whereas method versatility was more clearly associated with combined informal and professional awareness (RRR = 1.35, 95% CI [1.12, 1.63]). Interpersonal functions were associated with both professional-only awareness (RRR = 1.77, 95% CI [1.50, 2.08]) and combined informal and professional awareness (RRR = 1.72, 95% CI [1.53, 1.93]). Intrapersonal functions were not independently associated with either professional configuration (Table S2).

Within the 760 participants classified as having professional awareness, 251 (33.0%) reported mental-health-professional awareness without physician awareness, 319 (42.0%) reported physician awareness without mental-health-professional awareness, and 190 (25.0%) reported both. In a post hoc paired GEE model comparing these two professional recipient classes directly, the associations of depressive symptoms, method versatility, intrapersonal functions, and interpersonal functions did not differ significantly between mental-health-professional and physician awareness (all slope-difference *p* values ≥ 0.192; Table S5).

Excluding the 589 participants who reported that NSSI had required professional medical attention left 1807 participants (607 no assessed awareness, 639 informal-only awareness, and 561 professional awareness). In this subset, the direct multinomial professional-versus-informal-only contrasts remained positive for depressive symptoms (RRR = 1.46, 95% CI [1.27, 1.68], *p* < 0.001), method versatility (RRR = 1.25, 95% CI [1.03, 1.52], *p* = 0.026), and interpersonal functions (RRR = 1.27, 95% CI [1.12, 1.43], *p* < 0.001). In the paired GEE, the professional-versus-informal slope difference remained clear for depressive symptoms (OR = 1.59, 95% CI [1.37, 1.85], Holm-adjusted *p* < 0.001), but the slope differences for method versatility (OR = 1.17, 95% CI [0.95, 1.43], Holm-adjusted *p* = 0.132) and interpersonal functions (OR = 1.13, 95% CI [1.00, 1.28], Holm-adjusted *p* = 0.116) were attenuated (Table S6).

The interpersonal-function decomposition did not indicate that the overall association was simply a communication-item artifact. When the communication/signaling pair and the other three interpersonal items were entered simultaneously, the remaining interpersonal items were associated with informal-only versus no assessed awareness (RRR = 1.37, 95% CI [1.19, 1.57], *p* < 0.001) and professional versus no assessed awareness (RRR = 1.49, 95% CI [1.29, 1.73], *p* < 0.001). The communication/signaling pair was not associated with informal-only versus none (RRR = 1.03, 95% CI [0.90, 1.19], *p* = 0.624), but was associated with professional versus none (RRR = 1.21, 95% CI [1.05, 1.39], *p* = 0.009) and professional versus informal-only awareness (RRR = 1.17, 95% CI [1.02, 1.34], *p* = 0.029). Full estimates are reported in Table S7.

## 4. Discussion

NSSI known only to informal recipients differed from NSSI known to a professional. Depressive symptoms and method versatility were more strongly associated with professional awareness, whereas interpersonal-function endorsement characterized both informal and professional awareness. Intrapersonal functions did not contribute independently after adjustment. Among participants with lifetime NSSI, 33.9% reported that none of the five assessed recipient categories knew about the behavior. These are concurrent profiles. They do not identify how awareness arose or whether professional awareness was followed by assessment or treatment.

The associations were modest in magnitude. Unadjusted effect sizes were small for intrapersonal functions (η^2^ = 0.012) and small to modest for interpersonal functions (η^2^ = 0.051), and overall separation among recipient configurations was limited (McFadden R^2^ = 0.039). The results characterize group profiles; they do not support individual classification or clinical prediction.

### 4.1. NSSI Functions and Recipient Context

Interpersonal-function endorsement was associated with awareness by both informal and professional recipients. Several interpersonal items explicitly address communicating distress, obtaining care or support, and conveying the seriousness of distress, creating potential conceptual proximity to the awareness outcome. The post hoc decomposition, however, showed that the association was not reducible to the two communication/signaling items: the remaining interpersonal items retained associations with both informal-only and professional awareness relative to no assessed awareness. At the same time, the communication/signaling pair contributed to the direct professional-versus-informal-only contrast. These findings argue against treating the interpersonal composite as a simple proxy for communication, while still requiring caution because item content and recipient awareness are conceptually related. None of these cross-sectional associations establishes that a function prompted disclosure or recognition.

The multinomial and GEE results for interpersonal functions answer different questions and therefore are not contradictory. The multinomial model compares mutually exclusive recipient configurations; participants with both informal and professional awareness are included in the professional configuration, and the RRR of 1.25 compares that configuration with informal-only awareness. The paired GEE instead models any informal awareness and any professional awareness as two correlated binary outcomes, so participants with both types of awareness contribute a positive outcome to both recipient classes. Its slope ratio of 1.09 (95% CI [0.98, 1.22]) tests whether the interpersonal-function association itself is statistically stronger for professional than informal awareness and did not reach significance. The four-category model supports this distinction: interpersonal functions were positively associated with both professional-only and combined informal-professional configurations.

Intrapersonal functions followed a different pattern. Scores were higher in the professional-awareness group in unadjusted comparisons, but the association did not persist after depressive symptoms, interpersonal functions, and NSSI characteristics were included in the model. This finding concerns discrimination between recipient configurations; it should not be read as evidence that intrapersonal functions are unimportant to NSSI itself.

Recipient composition may account for part of the inconsistency across earlier disclosure studies. Armiento et al. [15] emphasized interpersonal influence, Mirichlis et al. [16] identified marking distress and anti-dissociation, and Ammerman et al. [17] reported broader intrapersonal correlates. Those studies also differed in measurement and covariate structure. Combining informal and professional recipients may therefore obscure recipient-specific patterns. Replication is needed in studies that measure recipient type, route of awareness, and timing directly.

### 4.2. Depressive Symptoms, Method Versatility, and Professional Awareness

Depressive symptoms and method versatility provided the clearest differentiation between professional and informal-only awareness in the primary analyses. Unlike the interpersonal-function items, neither variable contains communication or support content that is inherently close to the awareness outcome. Their temporal relation to awareness remains unknown. PHQ-8 scores referred to the preceding two weeks, whereas a professional may have learned about the NSSI months or years earlier. A broader repertoire of methods may increase opportunities for recognition or accompany a more complex NSSI history, but method count is not a measure of injury severity. In the medical-attention-excluded sensitivity analysis, the direct multinomial method-versatility contrast remained statistically significant, whereas the GEE recipient-class slope difference was attenuated and no longer significant. This attenuation may reflect reduced precision or a different case mix and should not be interpreted as evidence that medical recognition caused professional awareness. It does indicate that the recipient-specific method-versatility finding is less robust than the depressive-symptom finding.

The inverse association between depressive symptoms and informal-only versus no assessed awareness is specific to the multinomial comparison and should not be interpreted as evidence that depression inhibits disclosure to friends or family. After adjustment, higher depressive symptoms were associated with a lower relative likelihood of the informal-only configuration and a higher relative likelihood of professional awareness. The GEE analysis confirmed that the depressive-symptom association differed between recipient classes, but the cross-sectional design does not imply movement from one category to another.

### 4.3. Clinical Implications for Assessment and Care

Current self-harm guidance emphasizes psychosocial assessment of the functions of self-harm, current needs and safety, support networks, and the appropriate involvement of family or carers, while cautioning against reducing assessment to risk scores or prediction rules [1]. The present data add a practical question: who already knows about the NSSI, and what followed that awareness? One-third of participants reported that none of the five assessed recipient categories knew about their NSSI. Professional awareness, by contrast, did not establish that an NSSI assessment, suicide risk assessment, intervention, or continuing care had occurred.

Recipient awareness is contextual information for clinical formulation, not a proxy for severity, safety, or treatment need. Direct assessment of current NSSI, suicidal thoughts and behaviors, psychiatric symptoms, and immediate safety remains necessary regardless of who already knows about the behavior.

Informal recipients also warrant attention in clinical formulation because their responses may shape the safety of subsequent communication [10,12]. With the individual’s consent and within the limits of confidentiality, clinicians can ask what friends, family members, or partners know, how they responded, and whether the individual wants them involved in support or safety planning. We did not assess recipient reactions, referral processes, current suicide risk, or the timing of professional contact. The data cannot identify which informal responses facilitate professional engagement or improve outcomes; they show only that NSSI known solely within personal relationships had a different concurrent profile from NSSI known to a professional.

### 4.4. Population Context and Cultural Considerations

Arab female university students are rarely represented in recipient-specific NSSI research. Studies of help-seeking in Arab populations identify privacy, trust, stigma, family involvement, and service acceptability as potentially relevant concerns [18,19]. These factors may matter when adapting assessment and referral practices, but they were not measured here and cannot explain the observed coefficients. Without comparison groups, the findings cannot be characterized as culturally or gender specific. Future longitudinal studies should measure candidate cultural and service-use mechanisms directly, including anticipated and experienced stigma, confidentiality concerns, family norms, trust in services, perceived acceptability and accessibility of care, and recipient responses, and establish their timing relative to NSSI awareness and professional contact. Cross-group designs would additionally be needed to test whether such pathways differ across cultural contexts.

### 4.5. Strengths and Limitations

The large lifetime-NSSI subgroup permitted direct comparison of several recipient configurations and formal within-participant comparisons of informal and professional awareness. Robust standard errors, adjusted probabilities, GEE models, and sensitivity analyses reduced reliance on a single analytic specification. Maintaining the distinction between awareness and disclosure also helped align the interpretation with what the measures actually captured.

Several measurement features limit interpretation. The primary professional-awareness category combined mental health professionals and physicians, who may learn about NSSI through different routes, including clinical inquiry, visible injury, or medical treatment. The post hoc recipient-specific GEE did not identify significant slope differences between mental-health-professional and physician awareness for the focal covariates, and the medical-attention-excluded analysis preserved several primary contrasts, but these checks cannot establish how or when either professional became aware. We did not establish the route or timing of awareness, whether awareness preceded or followed the current symptom profile, whether an NSSI- or suicide-focused assessment occurred, or whether treatment was initiated. The awareness items also did not identify the first recipient or the response received. Professional awareness should therefore be understood as recipient status, not as a measure of access to or quality of care.

The cross-sectional design precludes temporal or causal conclusions about the relations among depressive symptoms, NSSI functions, NSSI characteristics, and recipient awareness. Awareness was assessed for five recipient categories only, so no assessed awareness does not imply complete concealment. The PHQ-8 captured symptoms during the preceding two weeks, whereas recipient awareness could have occurred at any point in the participant’s NSSI history. Some interpersonal-function items also overlap conceptually with communication and support, which may partly account for their association with awareness.

Digital and snowball recruitment limit generalizability, preclude calculation of a conventional response rate, and do not support population prevalence estimates. NSSI function scores were based on abbreviated ISAS-derived item sets rather than the complete subscales. Accordingly, they did not comprehensively sample the full range of functions represented in the ISAS; functions such as self-care, sensation seeking, peer bonding, toughness, revenge, autonomy, and boundary-related motives were not represented as complete functional domains. The domain scores should therefore be interpreted as study-specific abbreviated composites rather than exhaustive measures of intrapersonal or interpersonal functioning. All measures were self-reported, and the analyses were not preregistered. The additional peer-review sensitivity analyses were explicitly post hoc. Prospective studies should establish the timing and route of awareness and assess recipient responses, current suicidal thoughts and behaviors, clinical assessment, and subsequent service engagement.

## 5. Conclusions

Informal-only and professional awareness of NSSI were associated with different concurrent profiles. In the primary analyses, depressive symptoms and method versatility were more strongly associated with professional awareness, although the method-versatility slope difference was attenuated in the sensitivity analysis excluding NSSI that required medical attention. Interpersonal-function endorsement characterized both recipient classes and was not explained solely by communication/signaling items. For clinicians, knowing who is aware of NSSI may add context to formulation, but it is not a proxy for severity, safety, treatment engagement, or quality of care. Assessment should establish current NSSI, suicidal thoughts and behaviors, functions, support, and care needs directly. Longitudinal studies are needed to determine how routes of awareness and recipient responses relate to subsequent help-seeking and care.

## Figures and Tables

**Table 1:** Characteristics of the lifetime-NSSI subgroup by recipient-awareness configuration.

Characteristic	No Assessed Awareness (*n* = 812)	Informal-Only Awareness (*n* = 824)	Professional Awareness (*n* = 760)	*p*	Effect Size
Age, years	22.27 (3.92)	21.91 (3.39)	22.18 (3.68)	0.101	0.002
Partnered or married	324 (39.9%)	324 (39.3%)	309 (40.7%)	0.863	0.000
Previous mental-health treatment	196 (24.1%)	220 (26.7%)	219 (28.8%)	0.109	0.043
PHQ-8 total	13.58 (5.47)	12.82 (5.29)	15.03 (5.07)	<0.001	0.029
Lifetime NSSI frequency	44 [15, 107]	37 [13, 96]	55 [18, 143]	<0.001	0.008
Number of NSSI methods	3.45 (2.13)	3.26 (2.03)	3.94 (2.32)	<0.001	0.017
Intrapersonal functions	2.86 (1.00)	2.82 (0.96)	3.07 (0.97)	<0.001	0.012
Interpersonal functions	2.69 (0.95)	2.94 (0.94)	3.22 (0.92)	<0.001	0.051

Note. Values are M (SD), *n* (%), or median [interquartile range]. Effect sizes are η^2^ for continuous variables, ε^2^ for raw lifetime NSSI frequency, and Cramér’s V for categorical variables. Omnibus tests were Welch ANOVA, Kruskal–Wallis, or Pearson chi-square, as appropriate. Awareness refers only to the five assessed recipient categories.

**Table 2:** Primary Multinomial Model Of Recipient-Specific NSSI awareness.

Contrast/Covariate	RRR	95% CI	*p*
Informal-only vs. none			
Age	0.90	[0.81, 0.99]	0.030
Partnered or married	1.03	[0.84, 1.27]	0.751
PHQ-8 depressive symptoms	0.81	[0.72, 0.92]	<0.001
Log lifetime NSSI frequency	0.95	[0.81, 1.12]	0.557
NSSI method versatility	0.94	[0.80, 1.11]	0.494
Intrapersonal functions	1.07	[0.95, 1.21]	0.259
Interpersonal functions	1.39	[1.25, 1.54]	<0.001
Professional vs. none			
Age	0.98	[0.88, 1.08]	0.686
Partnered or married	1.00	[0.80, 1.23]	0.978
PHQ-8 depressive symptoms	1.14	[1.01, 1.30]	0.036
Log lifetime NSSI frequency	0.78	[0.65, 0.93]	0.006
NSSI method versatility	1.34	[1.13, 1.60]	<0.001
Intrapersonal functions	1.08	[0.95, 1.22]	0.250
Interpersonal functions	1.73	[1.55, 1.93]	<0.001
Professional vs. informal-only			
Age	1.09	[0.99, 1.21]	0.086
Partnered or married	0.96	[0.78, 1.19]	0.736
PHQ-8 depressive symptoms	1.41	[1.25, 1.60]	<0.001
Log lifetime NSSI frequency	0.82	[0.68, 0.98]	0.028
NSSI method versatility	1.42	[1.19, 1.70]	<0.001
Intrapersonal functions	1.00	[0.89, 1.14]	0.949
Interpersonal functions	1.25	[1.12, 1.38]	<0.001

Note. N = 2396. RRR = relative risk ratio. All RRRs are adjusted for the covariates shown. Continuous covariates were standardized within the lifetime-NSSI subgroup. HC3 robust standard errors were used. Previous mental-health treatment was omitted from the primary model and added in the sensitivity analysis reported in Table S1.

**Table 3:** Paired GEE tests of informal and professional awareness.

Covariate	Informal Awareness OR [95% CI]	Professional Awareness OR [95% CI]	Professional vs. Informal Slope OR [95% CI]	*p*
PHQ-8 depressive symptoms	0.83 [0.75, 0.92]	1.28 [1.15, 1.42]	1.54 [1.34, 1.75]	<0.001
NSSI method versatility	1.05 [0.91, 1.20]	1.38 [1.18, 1.61]	1.32 [1.09, 1.58]	0.007
Intrapersonal functions	1.09 [0.98, 1.20]	1.04 [0.93, 1.16]	0.96 [0.84, 1.09]	0.486
Interpersonal functions	1.34 [1.23, 1.46]	1.47 [1.34, 1.61]	1.09 [0.98, 1.22]	0.128

Note. N = 2396. GEE = generalized estimating equation. Each participant contributed correlated informal and professional awareness outcomes. Continuous covariates were standardized. The Holm correction was applied to the three focal slope-difference tests for depressive symptoms, method versatility, and interpersonal functioning. The intrapersonal-function test was descriptive and unadjusted.

## Data Availability

The data are not publicly available because they contain sensitive information concerning NSSI and suicidal thoughts and behaviors. Requests for de-identified data or analytic code may be considered by the corresponding author, subject to institutional and ethical approval.

## Supplementary Materials

The following supporting information can be downloaded at: https://www.mdpi.com/article/10.3390/jcm15208166/s1, Table S1: Robustness of the Primary Multinomial Associations Across Alternative Specifications; Table S2: Four-Category Recipient-Overlap Sensitivity Model; Table S3: Partner-Excluded GEE Comparison of Informal and Professional Awareness; Table S4: Complete Content of the Abbreviated NSSI Function Items Used in This Study; Table S5: Mental-Health-Professional and Physician Awareness: Recipient Composition and Paired GEE Comparison; Table S6: Sensitivity Analyses Excluding NSSI Requiring Professional Medical Attention; Table S7: Interpersonal-Function Decomposition Sensitivity Model.
