# Supplementary material for: Informal and Professional Awareness of Nonsuicidal Self-Injury: Clinical and Functional Correlates Among Arab Female University Students
Source: J Clin Med. 2026 Oct 20;15(20):8166. doi: 10.3390/jcm15208166 (PMC13705944; doi:10.3390/jcm15208166)
Supplement: Supplementary file 1 [file jcm-15-08166-s001.zip › jcm-4604953-supplementary.pdf]

Supporting Information

Informal and Professional Awareness of Nonsuicidal Self-Injury: Clinical and Functional Correlates Among Arab Female University Students

Sami Hamdan and Eyad Hallaq

Supplementary analytic framework

The supplementary analyses evaluate the robustness of the primary three-category multinomial model and paired GEE. All analyses used the lifetime-NSSI subgroup (N = 2,396). Continuous covariates were standardized within this subgroup. Robust standard errors were used for multinomial models and for GEE models.

Table S1

Robustness of the Primary Multinomial Associations Across Alternative Specifications

**Panel/specification**	**Contrast**	**Covariate**	**Adjusted RRR**	**95% CI**	**p**
A. Model adding previous treatment	Informal-only vs none	PHQ-8 depressive symptoms	0.81	[0.71, 0.91]	< .001
A. Model adding previous treatment	Informal-only vs none	NSSI method versatility	0.94	[0.80, 1.11]	.485
A. Model adding previous treatment	Informal-only vs none	Intrapersonal functions	1.07	[0.95, 1.21]	.251
A. Model adding previous treatment	Informal-only vs none	Interpersonal functions	1.39	[1.25, 1.54]	< .001
A. Model adding previous treatment	Informal-only vs none	Previous mental-health treatment/counseling	1.18	[0.94, 1.48]	.157
A. Model adding previous treatment	Professional vs none	PHQ-8 depressive symptoms	1.13	[1.00, 1.29]	.050
A. Model adding previous treatment	Professional vs none	NSSI method versatility	1.34	[1.13, 1.60]	.001

Panel/specification	Contrast	Covariate	Adjusted RRR	95% CI	p
A. Model adding previous treatment	Professional vs none	Intrapersonal functions	1.08	[0.95, 1.22]	.239
A. Model adding previous treatment	Professional vs none	Interpersonal functions	1.73	[1.55, 1.93]	< .001
A. Model adding previous treatment	Professional vs none	Previous mental-health treatment/counseling	1.22	[0.97, 1.55]	.090
A. Model adding previous treatment	Professional vs informal-only	PHQ-8 depressive symptoms	1.41	[1.24, 1.59]	< .001
A. Model adding previous treatment	Professional vs informal-only	NSSI method versatility	1.42	[1.19, 1.70]	< .001
A. Model adding previous treatment	Professional vs informal-only	Intrapersonal functions	1.00	[0.89, 1.14]	.944
A. Model adding previous treatment	Professional vs informal-only	Interpersonal functions	1.25	[1.12, 1.38]	< .001
A. Model adding previous treatment	Professional vs informal-only	Previous mental-health treatment/counseling	1.04	[0.83, 1.30]	.738
B. Informal awareness excludes partner	Informal-only vs none	PHQ-8 depressive symptoms	0.80	[0.71, 0.90]	< .001
B. Informal awareness excludes partner	Informal-only vs none	NSSI method versatility	0.98	[0.83, 1.15]	.801
B. Informal awareness excludes partner	Informal-only vs none	Intrapersonal functions	1.08	[0.95, 1.21]	.235
B. Informal awareness excludes partner	Informal-only vs none	Interpersonal functions	1.33	[1.20, 1.48]	< .001
B. Informal awareness excludes partner	Professional vs none	PHQ-8 depressive symptoms	1.14	[1.01, 1.29]	.031
B. Informal awareness excludes partner	Professional vs none	NSSI method versatility	1.37	[1.15, 1.63]	< .001
B. Informal awareness excludes partner	Professional vs none	Intrapersonal functions	1.08	[0.95, 1.22]	.248
B. Informal awareness excludes partner	Professional vs none	Interpersonal functions	1.68	[1.51, 1.86]	< .001
B. Informal awareness excludes partner	Professional vs informal-only	PHQ-8 depressive symptoms	1.43	[1.26, 1.62]	< .001
B. Informal awareness excludes partner	Professional vs informal-only	NSSI method versatility	1.40	[1.17, 1.67]	< .001
B. Informal awareness excludes partner	Professional vs informal-only	Intrapersonal functions	1.00	[0.88, 1.13]	.992

Panel/specification	Contrast	Covariate	Adjusted RRR	95% CI	p
B. Informal awareness excludes partner	Professional vs informal-only	Interpersonal functions	1.26	[1.13, 1.40]	< .001
C. Primary	Informal-only vs none	Log lifetime NSSI frequency	0.95	[0.81, 1.12]	.557
C. Primary	Informal-only vs none	NSSI method versatility	0.94	[0.80, 1.11]	.494
C. Primary	Professional vs none	Log lifetime NSSI frequency	0.78	[0.65, 0.93]	.006
C. Primary	Professional vs none	NSSI method versatility	1.34	[1.13, 1.60]	< .001
C. Primary	Professional vs informal-only	Log lifetime NSSI frequency	0.82	[0.68, 0.98]	.028
C. Primary	Professional vs informal-only	NSSI method versatility	1.42	[1.19, 1.70]	< .001
C. Frequency-only specification	Informal-only vs none	Log lifetime NSSI frequency	0.91	[0.82, 1.01]	.092
C. Frequency-only specification	Professional vs none	Log lifetime NSSI frequency	0.99	[0.89, 1.11]	.863
C. Frequency-only specification	Professional vs informal-only	Log lifetime NSSI frequency	1.08	[0.97, 1.21]	.160
C. Method-only specification	Informal-only vs none	NSSI method versatility	0.91	[0.82, 1.01]	.085
C. Method-only specification	Professional vs none	NSSI method versatility	1.11	[1.00, 1.24]	.055
C. Method-only specification	Professional vs informal-only	NSSI method versatility	1.22	[1.09, 1.36]	< .001

Note. Panel A adds previous mental-health treatment or counseling to the primary model. Panel B reconstructs informal awareness from friend and family awareness only. Panel C compares the concurrent frequency–method specification with models containing either lifetime frequency or method versatility alone. All models retained age, relationship status, PHQ-8 symptoms, intrapersonal functions, and interpersonal functions as applicable. Only estimates central to the robustness questions are shown.

Table S2

Four-Category Recipient-Overlap Sensitivity Model

Contrast	Covariate	Adjusted RRR	95% CI	p
Informal-only vs none	Age	0.90	[0.81, 0.99]	.031
Informal-only vs none	Partnered or married	1.03	[0.84, 1.27]	.751
Informal-only vs none	PHQ-8 depressive symptoms	0.81	[0.72, 0.92]	< .001
Informal-only vs none	Log lifetime NSSI frequency	0.95	[0.81, 1.12]	.558
Informal-only vs none	NSSI method versatility	0.94	[0.80, 1.11]	.494
Informal-only vs none	Intrapersonal functions	1.07	[0.95, 1.21]	.259
Informal-only vs none	Interpersonal functions	1.39	[1.25, 1.54]	< .001
Professional-only vs none	Age	0.90	[0.76, 1.06]	.222
Professional-only vs none	Partnered or married	1.32	[0.95, 1.84]	.096
Professional-only vs none	PHQ-8 depressive symptoms	1.47	[1.21, 1.80]	< .001
Professional-only vs none	Log lifetime NSSI frequency	0.71	[0.53, 0.95]	.022
Professional-only vs none	NSSI method versatility	1.32	[0.98, 1.77]	.067
Professional-only vs none	Intrapersonal functions	1.00	[0.83, 1.20]	.973
Professional-only vs none	Interpersonal functions	1.77	[1.50, 2.08]	< .001
Both informal and professional vs none	Age	1.01	[0.90, 1.12]	.912
Both informal and professional vs none	Partnered or married	0.90	[0.72, 1.14]	.385
Both informal and professional vs none	PHQ-8 depressive symptoms	1.05	[0.92, 1.20]	.467
Both informal and professional vs none	Log lifetime NSSI frequency	0.81	[0.67, 0.98]	.030
Both informal and professional vs none	NSSI method versatility	1.35	[1.12, 1.63]	.001
Both informal and professional vs none	Intrapersonal functions	1.10	[0.96, 1.27]	.157
Both informal and professional vs none	Interpersonal functions	1.72	[1.53, 1.93]	< .001

Note. The four mutually exclusive categories were no assessed awareness (n = 812), informal-only awareness (n = 824), professional-only awareness (n = 197), and combined informal and professional awareness (n = 563). No assessed awareness was the reference category. HC3 robust standard errors were used.

Table S3

Partner-Excluded GEE Comparison of Informal and Professional Awareness

Covariate	Professional vs informal slope OR	95% CI	Raw p	Holm-adjusted p
PHQ-8 depressive symptoms	1.52	[1.33, 1.74]	< .001	< .001
NSSI method versatility	1.30	[1.08, 1.57]	.006	.012
Interpersonal functions	1.15	[1.03, 1.28]	.015	.015

Note. Informal awareness was defined using friend and family awareness only. The slope OR compares the association with professional awareness against the association with informal awareness. Holm adjustment was applied to the three focal interaction tests.

Table S4

Complete Content of the Abbreviated NSSI Function Items Used in This Study

The table provides the English rendering of the item content administered in Arabic. Each item was rated from 1 (not at all) to 5 (extremely). These 10 items formed study-specific abbreviated composites and do not constitute the complete ISAS subscales.

Domain	Item	English rendering of item content
Intrapersonal	1	Relief from overwhelming distress
Intrapersonal	2	Self-punishment
Intrapersonal	3	Reduction of numbness or detachment
Intrapersonal	4	Making psychological pain concrete
Intrapersonal	5	Reduction of suicidal thoughts or prevention of suicidal action
Interpersonal	1	Communication of distress
Interpersonal	2	Obtaining care or support
Interpersonal	3	Conveying the seriousness of distress
Interpersonal	4	Influencing an interpersonal situation
Interpersonal	5	Preventing rejection or abandonment

Note. Item order follows the analytic variables used to construct the intrapersonal and interpersonal mean scores. The content is reported to make the abbreviated measurement transparent; it should not be interpreted as reproducing the full Inventory of Statements About Self-Injury.

Table S5

Mental-Health-Professional and Physician Awareness: Recipient Composition and Paired GEE Comparison

Panel A. Recipient composition within the professional-awareness group (n = 760)

Professional recipient configuration	n	% of professional-awareness group
Mental-health professional only	251	33.0
Physician only	319	42.0
Both mental-health professional and physician	190	25.0

Panel B. Paired GEE comparison of mental-health-professional and physician awareness

Covariate	Mental-health professional OR [95% CI]	Physician OR [95% CI]	Physician vs MHP slope OR [95% CI]	p
PHQ-8 depressive symptoms	1.29 [1.13, 1.47]	1.23 [1.09, 1.39]	0.96 [0.82, 1.11]	.559
NSSI method versatility	1.26 [1.04, 1.51]	1.33 [1.12, 1.59]	1.06 [0.85, 1.33]	.609
Intrapersonal functions	0.97 [0.85, 1.11]	1.08 [0.95, 1.22]	1.11 [0.95, 1.30]	.192
Interpersonal functions	1.34 [1.21, 1.49]	1.38 [1.24, 1.52]	1.02 [0.90, 1.16]	.717

Note. N = 2,396. Each participant contributed two correlated binary outcomes: awareness by a therapist/mental-health professional and awareness by a physician. Models included recipient class, age, relationship status, PHQ-8 symptoms, log lifetime NSSI frequency, method versatility, intrapersonal functions, interpersonal functions, and recipient-class-by-covariate interactions. Continuous covariates were standardized. Robust standard errors and an exchangeable working correlation were used. The slope OR tests whether the covariate association differed between physician and mental-health-professional awareness. These analyses were post hoc.

Table S6

Sensitivity Analyses Excluding NSSI Requiring Professional Medical Attention

Participants who reported that an NSSI episode had required professional medical attention were excluded. The retained analytic sample was N = 1,807: no assessed awareness n = 607, informal-only awareness n = 639, professional awareness n = 561.

Covariate	Multinomial: professional vs informal-only RRR [95% CI]	p	GEE: professional vs informal slope OR [95% CI]	Adjusted p
PHQ-8 depressive symptoms	1.46 [1.27, 1.68]	< .001	1.59 [1.37, 1.85]	< .001
NSSI method versatility	1.25 [1.03, 1.52]	.026	1.17 [0.95, 1.43]	.132
Intrapersonal functions	1.00 [0.87, 1.16]	.953	0.95 [0.82, 1.10]	.495*
Interpersonal functions	1.27 [1.12, 1.43]	< .001	1.13 [1.00, 1.28]	.116

Note. RRR = relative risk ratio; GEE = generalized estimating equation. The same covariate structure as the primary models was used, with continuous covariates re-standardized within the retained subset. Holm adjustment was applied to the three focal GEE slope-difference tests (PHQ-8 symptoms, method versatility, and interpersonal functions). *The intrapersonal-function GEE p value is descriptive and unadjusted. These analyses were post hoc.

Table S7

Interpersonal-Function Decomposition Sensitivity Model

The interpersonal composite was decomposed into a communication/signaling pair (communication of distress; conveying the seriousness of distress) and the remaining three interpersonal items (obtaining care or support; influencing an interpersonal situation; preventing rejection or abandonment). The two standardized components were entered simultaneously in the primary multinomial model.

Interpersonal component	Informal-only vs none RRR [95% CI]	p	Professional vs none RRR [95% CI]	p	Professional vs informal-only RRR [95% CI]	p
Communication/signaling pair	1.03 [0.90, 1.19]	.624	1.21 [1.05, 1.39]	.009	1.17 [1.02, 1.34]	.029
Other interpersonal items	1.37 [1.19, 1.57]	< .001	1.49 [1.29, 1.73]	< .001	1.09 [0.95, 1.26]	.220

Note. N = 2,396. Both interpersonal components were included concurrently with age, relationship status, PHQ-8 symptoms, log lifetime NSSI frequency, method versatility, and intrapersonal functions. Continuous covariates were standardized within the lifetime-NSSI subgroup. HC3 robust standard errors were used. These analyses were post hoc and were conducted to assess whether the interpersonal-function association was attributable mainly to communication/signaling item content.
